# Efficacy of a transdiagnostic, prevention-focused program for at-risk young adults: a waitlist-controlled trial

**DOI:** 10.1017/S0033291722000046

**Published:** 2023-06

**Authors:** Nicole R. DeTore, Lauren Luther, Wisteria Deng, Jordan Zimmerman, Logan Leathem, Anne S. Burke, Maren B. Nyer, Daphne J. Holt

**Affiliations:** 1 Department of Psychiatry, Massachusetts General Hospital, Boston, MA, USA; 2 Harvard Medical School, Boston, MA, USA; 3 Department of Psychology, University of Georgia, Athens, GA, USA; 4 University of California, Los Angeles, CA, USA

**Keywords:** At-risk youth, depression, mindfulness, prevention, psychotic experiences, resilience, self-compassion, transdiagnostic

## Abstract

**Background:**

Prevention programs that are ‘transdiagnostic’ may be more cost-effective and beneficial, in terms of reducing levels of psychopathology in the general population, than those focused on a specific disorder. This randomized controlled study evaluated the efficacy of one such intervention program called Resilience Training (RT).

**Methods:**

College students who reported mildly elevated depressive or subclinical psychotic symptoms (‘psychotic experiences' (PEs)) (*n* = 107) were randomized to receiving RT (*n* = 54) or to a waitlist control condition (*n* = 53). RT consists of a four-session intervention focused on improving resilience through the acquisition of mindfulness, self-compassion, and mentalization skills. Measures of symptoms and these resilience-enhancing skills were collected before and after the 4-week RT/waitlist period, with a follow-up assessment 12-months later.

**Results:**

Compared to the waitlist control group, RT participants reported significantly greater reductions in PEs, distress associated with PEs, depression, and anxiety, as well as significantly greater improvements in resilience, mindfulness, self-compassion, and positive affect, following the 4-week RT/waitlist period (all *p* < 0.03). Moreover, improvements in resilience-promoting skills were significantly correlated with symptom reductions (all *p* < 0.05). Lastly, the RT-related reductions in PEs and associated distress were maintained at the 12-month follow-up assessment.

**Conclusions:**

RT is a brief, group-based intervention associated with improved resilience and reduced symptoms of psychopathology, with sustained effects on PEs, in transdiagnostically at-risk young adults. Follow-up studies can further assess the efficacy of RT relative to other interventions and test whether it can reduce the likelihood of developing a serious mental illness.

## Introduction

Numerous studies have shown that a portion of the risk for developing many serious mental illnesses is related to environmental factors, including stressful life events (Mandelli, Petrelli, & Serretti, 2015; Stilo et al., 2017; van Os, Kenis, & Rutten, 2010). Such environmental effects on mental illness risk are considered ‘modifiable risk factors’, which can be influenced by changes in the at-risk person's environment or in their responses to environmental stressors (Osborne, Willroth, DeVylder, Mittal, & Hilimire, 2017). Consistent with this model, studies have shown that levels of emotional ‘resilience’, which has been defined as the ability to adapt to or recover from stressful events (Rutter, 1985), can be increased by learning certain habits of thinking or emotion regulation skills (Choi, Stein, Dunn, Koenen, & Smoller, 2019; Hjemdal, Vogel, Solem, Hagen, & Stiles, 2011). For example, mindfulness practice may provide some protection for those who may be vulnerable to developing psychiatric disorders such as depression, attention-deficit hyperactivity disorder, and addiction (Tang & Leve, 2016).

Further supporting this concept are studies suggesting that intervening in at-risk populations during early or premorbid illness stages may prevent the development of psychiatric illnesses (Horowitz & Garber, 2006; McGorry et al., 2009; Mei et al., 2021; Stice, Shaw, Bohon, Marti, & Rohde, 2009), or at least delay the onset or lessen the severity of these illnesses when they occur (Addington & Heinssen, 2012; Mendelson & Eaton, 2018). However, to date, there are few evidence-based programs implemented in community settings that aim to reduce the impact of stressful life events and/or decrease subthreshold symptomatology, an established risk factor for the development of a range of psychiatric conditions (Docherty et al., 2020; Kelleher & Cannon, 2011; Upthegrove, Marwaha, & Birchwood, 2016), in at-risk populations. This may be in part because the evidence for the efficacy of interventions aiming to prevent the onset of serious mental illness in help-seeking at-risk individuals has been mixed (Fusar-Poli et al., 2020; Mei et al., 2021), suggesting that new approaches should be considered (Anglin, Galea, & Bachman, 2020; Fusar-Poli, 2017). One strategy that has been proposed (Dozois, Seeds, & Collins, 2009; McGorry, Hartmann, Spooner, & Nelson, 2018) is a transdiagnostic approach focused on less severe and less differentiated stages of psychopathology than what has been primarily targeted thus far.

The majority of prior prevention studies in psychiatry have not taken this approach but have focused on reducing risk for a particular illness (e.g. schizophrenia, depression, anxiety disorders) or group of related illnesses (e.g. depression and anxiety). However, there is increasing evidence that many neuropsychiatric conditions share biological (Anttila et al., 2016, 2018; Caspi & Moffitt, 2018) and environmental (Conway, Raposa, Hammen, & Brennan, 2018; Vachon, Krueger, Rogosch, & Cicchetti, 2015) risk factors and respond to similar types of interventions (Barlow et al., 2017; Newby, McKinnon, Kuyken, Gilbody, & Dalgleish, 2015). Thus, a convergent body of data suggests that interventions focused on individuals who may be at risk for a range of psychiatric illnesses may have large benefits.

Based on this literature, we developed and piloted an intervention, called Resilience Training (RT) (Burke et al., 2020), for young adults who carry some transdiagnostic risk for psychiatric illness, due to having mild-to-moderate symptoms of depression (which broadly increases the risk for developing psychiatric disorders (Balázs et al., 2013; Upthegrove et al., 2016)) and/or mild subclinical psychotic or psychotic-like symptoms, sometimes called ‘psychotic experiences' (PEs) (i.e. delusional ideas or perceptual aberrations associated with a small increase in risk for serious mental illnesses (Dominguez, Wichers, Lieb, Wittchen, & van Os, 2011; Kelleher & Cannon, 2011; Loewy, Pearson, Vinogradov, Bearden, & Cannon, 2011)). Depression and PEs frequently coexist in young people (Varghese et al., 2011), and PEs can be harbingers of more serious psychiatric conditions, with up to a 15-fold increase in risk (Poulton et al., 2000). This risk level associated with PEs is highest if the PEs are distressing or persistent (Kelleher et al., 2013), and these symptoms often increase in severity in response to life stressors and environmental risk factors for serious mental illness, such as cannabis abuse, urban living, and discrimination (Barkus, Morrison, Di Forti, & Murray, 2016; Krabbendam, 2005; Myin-Germeys & van Os, 2007; Read, Os, Morrison, & Ross, 2005; van Os, 2013). Thus, people with these symptoms typically show a vulnerability to the effects of stress, which may be modifiable to some extent in certain individuals (Broekman, 2011; Ozbay et al., 2007). Thus, the goal of RT is to enhance the ability of young people showing vulnerability to mental illness to withstand or adapt to the challenges of daily life (Masten, 2011) and major adverse events.

Because the modal age of onset for many serious mental illnesses is between the ages of 18 and 25 years of age (Häfner et al., 1994), which largely coincides with the age range of the majority of college students, the RT program was developed for young adults attending college. To reduce the effects of the stigma associated with mental health interventions, RT was advertised as a resilience-enhancing workshop rather than psychotherapy, and the RT workshops were held in classrooms or conference rooms on campus. The intervention uses a strengths-based framework and consists of four 1.5-hour-long sessions that focus on three evidence-based skills shown to improve mental health: mindfulness (Galante et al., 2018; Potes et al., 2018), mentalization (Bateman & Fonagy, 2013), and self-compassion (Neff & Germer, 2013). Prior studies have shown that these skills decrease various forms of emotional distress, such anxiety and depression (Frostadottir & Dorjee, 2019; Hayden, Müllauer, Gaugeler, Senft, & Andreas, 2018; Hofmann & Gómez, 2017; Neff & Germer, 2013) and improve emotion regulation and social functioning (Burke et al., 2020; Lindsay, Young, Brown, Smyth, & Creswell, 2019). A single-arm pilot trial of RT (Burke et al., 2020), conducted with 63 at-risk college students, demonstrated that the workshop was feasible and acceptable, with 91% of students attending 75% or more of the sessions. RT also led to significant pre-post reductions in PEs, depression, and anxiety, as well as improvements in resilience-related capacities.

To follow up this initial evidence for the feasibility and potential efficacy of RT, the current study aimed to test the efficacy of RT in increasing resilience and reducing symptoms of psychopathology in transdiagnostically at-risk college students, using a randomized waitlist-controlled design. This trial served as an intermediate step between our one-armed pilot study (Burke et al., 2020) and a future randomized controlled comparison with another psychological intervention. We hypothesized that those who participated in RT (compared to those randomized to the waitlist arm) would show a greater decrease in symptoms (PEs, depression, and anxiety) and a greater increase in overall resilience and the three resilience-related capacities (mindfulness, self-compassion, and mentalization) targeted by RT.

## Methods

### Overall design and procedures

Recruitment for this project occurred in two phases. The first phase was part of a larger longitudinal screening study examining factors that influence the trajectory of mental health symptoms in college students. For the screening study, participants were recruited from in-person mental health screenings conducted at both a large university and a liberal arts college in the metro Boston area. Three mental health screenings were conducted at each school. Each screening took place over 1 day, with booths set up in a high traffic area of the campus. Screening day advertisements consisted of signs describing the event as a ‘free psychological screening,’ and study staff were available during the screening to provide a brief overview of the study and answer any questions. Students who chose to participate in the longitudinal screening study signed informed consent, provided personal health history (whether they have been/are currently receiving therapy or taking psychotropic medications), and completed a battery of questionnaires. This battery included the Beck Depression Inventory (Beck, 1961) and the Peters et al., Delusions Inventory (Peters, Joseph, Day, & Garety, 2004) (see Measures below for full battery). Participants were compensated $20 for completing the screening.

The second phase of recruitment involved (1) identifying participants who were eligible to participate in the randomized controlled trial (RCT) study, and then (2) inviting these students to participate in the RCT. Participants were eligible for the RCT if they: (1) were between 18 and 30 years old, (2) were enrolled in an undergraduate program, and (3) endorsed mild to moderate depressive symptoms (BDI total score >5) and/or PEs (PDI total score >3). Following our prior procedures (Burke et al., 2020), we determined these cut-off scores based on median values identified in similar cohorts (DeCross et al., 2020; Farabaugh et al., 2012; Varghese et al., 2011). Given that we aimed to focus on those at-risk for mental illness, individuals actively engaged in mental health treatment (i.e. receiving treatment with psychotropic medications, other than medications used for treating attention deficit disorder and related conditions, or psychotherapy) were not eligible to participate. Eligible participants were then provided an overview of the RCT, and interested participants completed a second consent form for the RCT study. Participants were then randomized (using math.random in JavaScript) to: (1) the RT group or (2) a Waitlist (WL) group which received RT after 4 weeks.

To randomize the assignment of the participants to one of the two groups, the math.random function of JavaScript was used to generate random numbers that were distributed across the two groups. A member of the study staff then assigned incoming participants a number which determined their intervention group (RT vs WL). The waitlist design was chosen to best meet the needs of all the at-risk students, providing support during that same school semester to all of the students enrolled in the RCT. At the end of the 4-week waitlist period, the WL group participants completed an additional assessment battery (i.e. a second baseline assessment, which consisted of the same measures completed at the screening). This second baseline assessment doubled as the final waitlist period scores and as baseline scores prior to the WL group beginning RT. Participants were compensated $20 for completing each assessment and for attending each RT session. All procedures were approved by the Mass General Brigham Institutional Review Board (IRB) and the IRB of each of the participating institutions.

To identify a minimum recruitment target/sample size, we conducted a power analysis using the effect sizes of our pilot trial of RT (Burke et al., 2020). We estimated that the minimum sample size that would be sufficient to detect at least small-medium effect sizes (power = 0.80; significance = 0.01; *f* = 0.20) was 78 participants. Also, based on our pilot trial and the waitlist design, we estimated there would be approximately 20% attrition, resulting in a need for a minimum total recruitment sample size of 98.

### Resilience Training

RT consisted of four weekly 1.5-h group sessions, co-led by two faciliators, who were either a doctoral-level psychologist, psychiatrist, or an advanced doctoral student in clinical psychology. Briefly, RT begins by introducing the concept of resilience and that it can be modified (Session 1); it then focuses on teaching resilience-enhancing skills via didactic and experiential materials adapted from established mindfulness (Session 1), self-compassion (Session 2), and mentalization (Session 3) interventions and how these concepts and skills can be applied in daily life (Session 4). Each session involves introducing the skill and completing an experiential exercise as well as assigning a home practice relevant to the new skill. See Burke et al. (2020) for additional details about RT.

The sessions were audiotaped to enable an independent rating of fidelity to the RT program. Fifty percent of the sessions were rated using a nine-item adapted version of the mindfulness-based cognitive therapy adherence scale (Segal, Teasdale, Williams, & Gemar, 2002). Items such as, ‘To what extent did the therapists elicit feedback’ were rated on a 3-point scale (0 = no evidence of item, 1 = slight evidence, 2 = definite evidence). Fidelity ratings were completed by two psychologists who were familiar with the intervention but were not group leaders. To establish inter-rater reliability, 25% of the sessions were first double coded (intra-class correlation was 0.86 (*p* < 0.001)). Ratings indicated that there was a high level of fidelity to the RT program, with mean ratings of 1.93 (s.d. = 0.11; range = 1.63–2.00).

### Measures

We assessed demographic characteristics, psychiatric treatment information, symptoms and resilience factors in all participants. Brief measure descriptions are below, with additional details provided in the Online Supplemental Materials.

#### Symptoms

Depressive symptoms were assessed with the 21-item self-report Beck Depression Inventory – 1A [BDI – 1A; (Beck, 1961)]. Psychotic experiences (PEs), namely delusional beliefs and unusual experiences, were assessed with the self-report Peters Delusions Inventory [PDI; (Peters, Joseph, & Garety, 1999; Peters et al., 2004)]. The PDI has been well-validated in college samples (Fonseca-Pedrero, Paino, Santarén-Rosell, Lemos-Giráldez, & Muñiz, 2012) and uses non-stigmatizing and non-clinical language to describe common delusional experiences (e.g. paranoia, grandiosity, ideas of reference) and perceptual aberrations (e.g. experiences of being controlled; thought insertion, withdrawal, and echoing). Following our prior methods (Burke et al., 2020), we focused on the number of endorsed PEs (PDI total score) and the level of associated PE-related distress reported (PDI-Distress subscale score). Anxiety symptoms were measured with the 20-item state score of the self-report Spielberger State-Trait Anxiety Inventory [STAI; (Spielberger, Gorsuch, Lushene, Vagg, & Jacobs, 1983)].

#### Resilience factors

Resilience was assessed using the 25-items self-report Connor-Davidson Resilience scale [CD-RISC; (Connor & Davidson, 2003)]. Self-compassion was measured using the total score of the 26-item self-report Self Compassion Scale [SCS; (Neff, 2003)]. Mindfulness was measured using the total score of the Five Facet Mindfulness Questionnaire [FFMQ; (Baer, Smith, Hopkins, Krietemeyer, & Toney, 2006)]. As in Burke et al., 2020, a capacity related to mentalization, empathy, was measured using the Empathic Concern (EC) and Perspective Taking (PT) subscales (measuring the affective and cognitive components of empathy, respectively) of the self-report Interpersonal Reactivity Index [IRI; (Davis, 1983)]. Finally, positive affect was measured with the self-report Positive and Negative Affect Scale [PANAS; (Watson, Clark, & Tellegen, 1988)].

### Analyses

Data analyses were conducted in several steps. First, we conducted a series of *t* tests and chi-squared tests to assess comparability between the two randomized groups at baseline with respect to demographic variables, as well as depressive symptoms and PEs. For the remaining analyses assessing the impact of RT, we used an intention-to-treat analysis (Gupta, 2011), including all participants who were randomized regardless of RT attendance. To identify whether RT led to greater changes in the symptom and resilience-related outcomes than the WL period alone, we conducted a series of repeated measures analysis of variances (rmANOVA) with time (baseline *v.* second assessment scores) as the within-subjects factor, and group (RT *v.* WL) as the between-subjects factor. For participants in the WL group, their pre-intervention assessments were used in these analyses. Significant group × time interactions suggested the presence of an intervention effect and were followed up with post hoc independent-samples *t-*tests of change scores (4-week assessment – baseline) to elucidate group effects.

Next, as an exploratory follow-up analysis, we examined whether the same pattern of findings was present in the WL condition after they received RT. Specifically, we used paired-sample *t-*tests to examine whether there were significant changes in symptoms and resilience factors between their pre-intervention (i.e. pre-RT scores) and post-RT assessment scores. If the pattern of findings was similar, we planned to combine the groups to examine whether changes in symptoms and resilience factors persisted or emerged 12 months after receiving RT.

To identify whether changes in our RT intervention targets were associated with changes in symptoms, we conducted Pearson's correlations between significant changes (Post-RT – Pre-RT) in resilience factors and symptoms. All analyses were conducted with SPSS version 26.0.

## Results

### Screening and participant characteristics

**Fig. 1.**
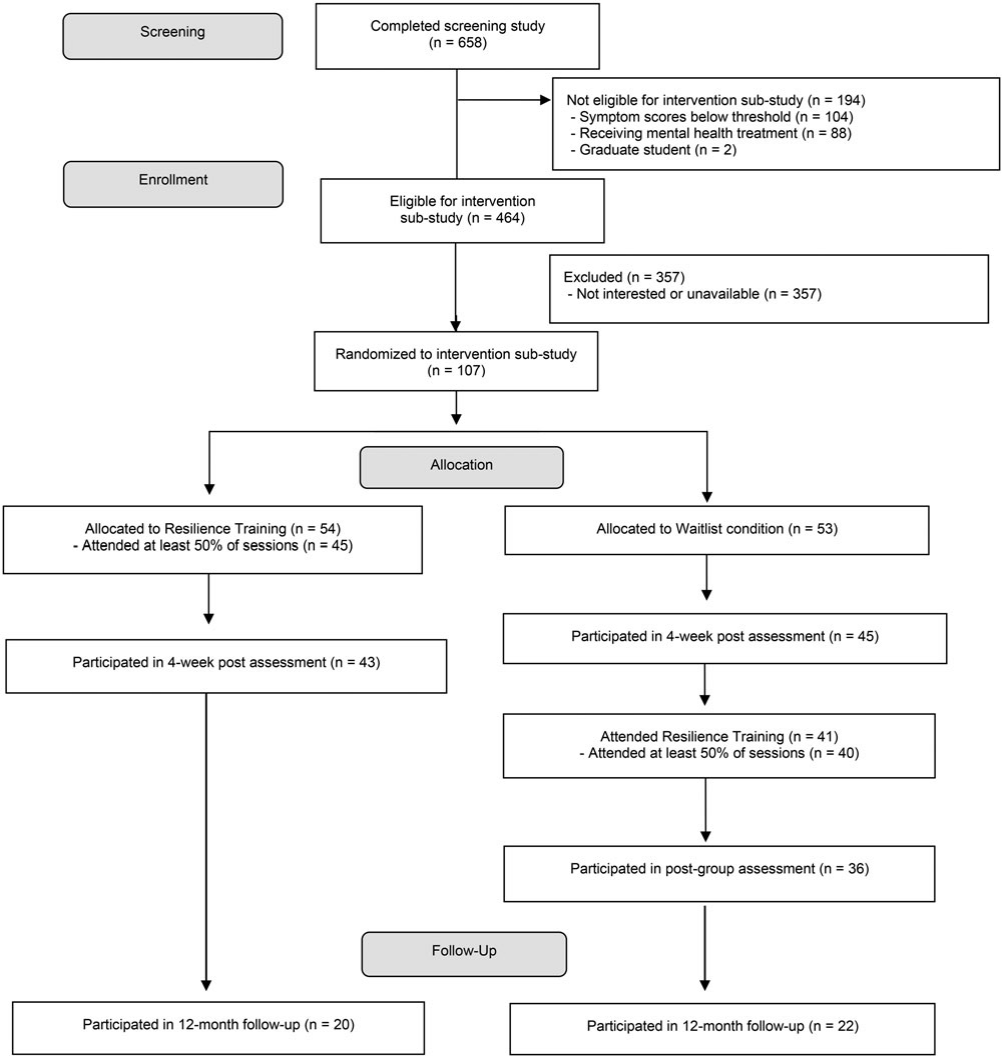
Consort flow diagram.

Six hundred and fifty-eight young adults completed the on-campus screenings. Participants had a mean age of 18.82 (s.d. = 1.12) and the majority were female (*n* = 422, 64.3%), Caucasian (*n* = 362, 55.2%), and Freshman (*n* = 426, 64.9%). Of those who completed the screening, 464 were eligible to participate in the RCT portion of the study. Of these, 293 were eligible based on both their PDI and BDI scores, 111 based on their PDI score alone, and 60 based on their BDI score alone. Among the 464 eligible students, 107 were interested in and available for the intervention groups; 77 of these were eligible based on both their PDI and BDI scores, 16 based on the PDI alone, and 14 based on the BDI alone. Many participants noted schedule conflicts as the main barrier to participation (the RT sessions were offered at 1–2 times during the week at each school). Participants were randomized to either the RT group (*n* = 54) or the WL group (*n* = 53). After the WL period, 41 participants in the WL group received RT. Across both conditions, 85 participants (*n* = 45 RT, *n* = 40 WL) attended at least 50% of the RT workshops. See Fig. 1 for the CONSORT diagram.

At baseline, the two groups did not significantly differ on any participant demographic variable or levels of depression or PEs (see Table 1). Those who were eligible for RT but were not randomized (*n* = 357) did not significantly differ on demographic variables or levels of depression or PEs from those who were randomized to the RT group (*n* = 107).

**Table 1.** Demographics and baseline symptoms by group for randomized participants

Variable	Resilience Training (*n* = 54)	Waitlist (*n* = 53)	Comparison statistic
	*n*, %	*n*, %	
Gender (*n*, % Female)	35, 64.8%	41, 77.4%	*χ*^2^ (1) = 2.05, *p* = 0.15
Race^a^			*χ*^2^ (3) = 0.77, *p* = 0.86
African American	5, 9.4%	4, 7.5%	
Asian	16, 30.2%	16, 30.2%	
Caucasian	27, 50.9%	30, 56.6%	
Other	5, 9.4%	3, 5.7%	
Ethnicity (*n*, % Hispanic)	9, 16.7%	3, 5.7%	*χ*^2^ (1) = 3.25, *p* = 0.07
Born in the U.S.	42, 77.8%	43, 81.1%	*χ*^2^ (1) = 0.18, *p* = 0.67
Year in School			*χ*^2^ (3) = 1.09, *p* = 0.78
Freshman	36, 66.7%	31, 58.5%	
Sophomore	10, 18.5%	13, 24.5%	
Junior	7, 13.0%	7, 13.2%	
Senior	1, 1.9%	2, 3.8%	
	M (s.d.)	M (s.d.)	
Mean parental education	15.8 (3.1)^b^	15.8 (2.6)	*t*(103) = −0.14, *p* = 0.89
BDI-I— Depressive symptoms	12.3 (8.3)	12.8 (7.0)	*t*(105) = −0.37, *p* = 0.71
PDI-Total – Psychotic experiences	6.8 (3.6)	7.4 (3.3)	*t*(105) = −0.90, *p* = 0.37

BDI, Beck Depression Inventory; PDI, Peters Delusion Inventory.

a Missing data for one participant.

b Missing data for two participants.

### Resilience Training v. waitlist outcomes

#### Symptoms

**Fig. 2.**
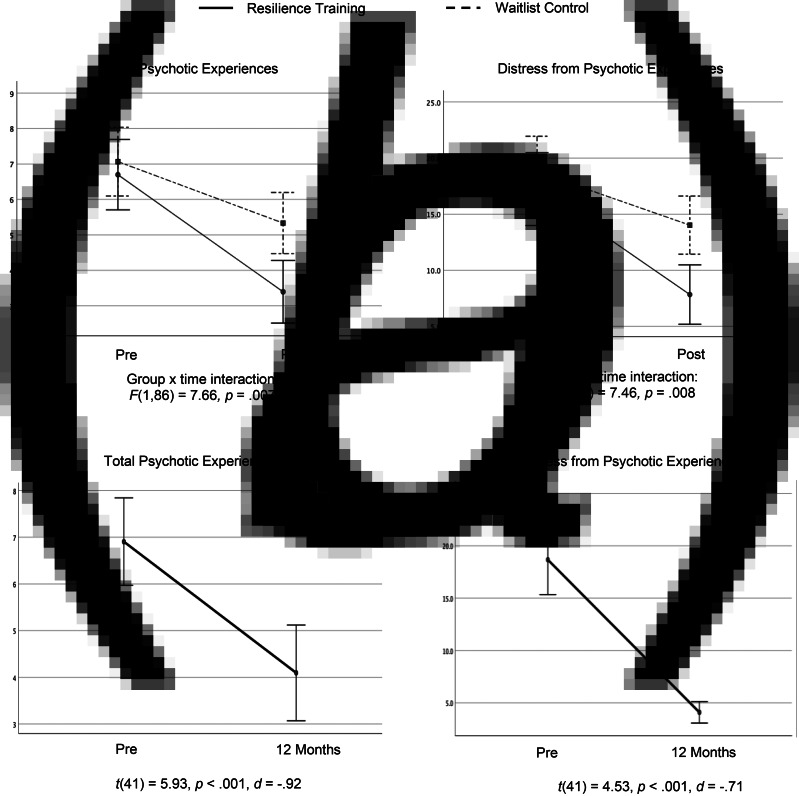
(*a*). Changes in psychotic experiences and related distress in the Resilience Training (*n* = 43) *v.* the Waitlist Control condition (*n* = 45). (*b*) Changes in psychotic experiences and related distress in those who completed Resilience Training at the 12 month follow-up time point (*n* = 42).

The change from baseline to 4 weeks was significantly different between the RT and WL groups (i.e. there was a significant group by time interaction) for depression [*F*(1,86) = 13.62, *p* < 0.001, *η_p_*^2^ *=* 0.14], PEs [*F*(1,86) = 7.66, *p* = 0.007, *η_p_*^2^ = 0.08], PE related distress [*F*(1,86) = 7.46, *p* = 0.008, *η_p_*^2^ *=* 0.08], and state anxiety [*F*(1,80) = 5.86, *p* = 0.02, *η_p_*^2^ *=* 0.07]. Specifically, compared to the WL group, participants in the RT group showed a greater decrease in depression [*t*(86) = −3.69, *p* < 0.001, *d* = −0.78], PEs [*t*(86) = −2.77, *p* = 0.007, *d* = −0.58], PE-related distress [*t*(86) = −2.73, *p* = 0.008, *d* = −0.58] (see Fig. 2a), and state anxiety [*t*(80) = −2.42, *p* = 0.02, *d* = −0.53].

#### Resilience factors

From baseline to 4 weeks, the change was significantly different between the RT and WL groups for resilience [*F*(1,46) = 13.23, *p* = 0.001, *η_p_*^2^ = 0.22], mindfulness [*F*(1,46) = 8.32, *p* = 0.006, *η_p_*^2^ = 0.15], self-compassion [*F*(1,46) = 8.28, *p* = 0.006, *η_p_*^2^ = 0.15], and positive affect [*F*(1,80) = 5.11, *p* = 0.03, *η_p_*^2^ = 0.06] (see Online Supplementary Table S1). Among these factors, the RT group participants demonstrated a greater increase in resilience [*t*(46) = 3.64, *p* = 0.001, *d* = 1.04], mindfulness [*t*(46) = 2.89, *p* = 0.006, *d* = 0.79], self-compassion [*t*(46) = 2.88, *p* = 0.006, *d* = 0.82], and positive affect [*t*(80) = 2.26, *p* = 0.03, *d* = 0.48] than the WL group. There were no significant group by time interactions for empathetic concern [*F*(1,47) = 0.57, *p* = 0.46] or perspective taking [*F*(1,47), = 0.004, *p* = 0.95].

### Pre-post Resilience Training outcomes

#### Waitlist group

A similar pattern of findings was observed when we examined changes in symptoms and resilience factors in the WL condition alone after they received RT (see Online Supplementary Table S2). Specifically, when comparing their second baseline to their post-RT group assessment, the WL group showed significant reductions in depression, PE-related distress, and anxiety (all *p* < 0.002). The WL group also showed significant improvements in resilience, mindfulness, self-compassion, and positive affect (all *p* < 0.042). There were no significant changes observed in empathic concern or perspective taking (*p* > 0.19).

#### 12-Month follow-up

Given that the change in scores from pre-RT to post-RT was highly similar for both the RT and WL groups, we combined the two samples to examine the impact of RT at 12 months following completion of RT. When comparing pre-RT scores to 12-month follow-up scores, the significant reduction in PEs [*t*(41) = 5.93, *p* < 0.001, *d* = −0.92] and PE related distress [*t*(41) = 4.53, *p* < 0.001, *d* = −0.71] was maintained (see Fig. 2b). No other symptoms or any resilience factor studied showed significant changes between pre-RT and 12-month scores (see Online Supplementary Table S3).

### Associations between changes in resilience factors and symptoms

In both groups, greater improvements after receiving RT in resilience, mindfulness, and self-compassion were significantly associated with greater reductions in depression, PE related distress (but not the number of PEs), and anxiety symptoms (see Table 2). Also, a greater increase in positive affect was significantly associated with a greater reduction in anxiety (*r* = −0.420, *p* < 0.001).

**Table 2.** Correlations between changes in resilience factors and symptoms after receiving RT

Change in resilience factors	Change in symptoms			
	Depression symptoms	Psychotic experiences	Psychotic experiences—distress	Anxiety symptoms
Resilience	−0.37*	−0.27	−0.44**	−0.35*
Mindfulness	−0.45**	−0.19	−0.37*	−0.51**
Self-compassion	−0.40**	−0.12	−0.35**	−0.55**
Positive affect	−0.16	0.10	−0.06	−0.42**

*p < 0.05; **p < 0.01.

## Discussion

This study demonstrated that a transdiagnostic intervention, Resilience Training (RT), was associated with significantly greater improvements in at-risk young adults in multiple symptoms of psychopathology that can be precursors of serious mental illness, including PEs and distress related to those symptoms, depression, and anxiety, compared to a waitlist control condition. Moreover, the significant reduction in PEs and the related distress was maintained 12 months after the 4-week RT intervention. These results are in line with those of our previous single-arm study (Burke et al., 2020) which found that those who received RT had significant post-treatment reductions in depression, anxiety, and PEs.

The skills or capacities targeted by the RT intervention such as self-compassion and mindfulness were also largely improved across both this current study and the previous single-arm study of RT, suggesting that RT is successfully targeting these resilience-enhancing mechanisms. Specifically, in the current study, participants receiving RT demonstrated greater increases in self-compassion and mindfulness, with large effect sizes. We also observed that greater changes in resilience, self-compassion, and mindfulness were significantly associated with greater reductions in depression, distress from PEs, and anxiety symptoms. These findings suggest that mindfulness and self-compassion are key mechanisms underlying the observed symptom improvement with RT. Although not directly tested, these results also align with the RT intervention model, which suggests that targeting these skills can lead to increases in resilience and, in turn, symptom improvement. Further, these results are consistent with work showing that targeting mindfulness and self-compassion can lead to reductions in anxiety and depression (Frostadottir & Dorjee, 2019; Hayden et al., 2018; Hofmann & Gómez, 2017; Neff & Germer, 2013). Similarly, these results extend prior studies showing that improved resilience is associated with lower depression and anxiety in both non-clinical samples and in individuals with psychosis (Beutel, Glaesmer, Wiltink, Marian, & Brähler, 2010; Rossi et al., 2017; Torgalsbøen, 2012).

Notably, consistent with our prior study (Burke et al., 2020), we did not observe significant changes in affective or cognitive aspects of empathy, our indirect measures of mentalization. This could suggest that mentalization is a less critical component of RT or that these indirect measures of mentalization-related skills, aspects of empathy, do not capture the mentalization skills that were taught in RT. Additional study of the mentalization portion of the RT intervention is warranted to determine whether it should be further enhanced and/or assessed using a more direct measure of mentalization.

Interestingly, the RT-related reduction in the number of PEs was not significantly associated with any of the improvements in the resilience-related factors, while the distress associated with PEs was. This finding is consistent with studies showing that distress related to PEs has a stronger relationship to clinical outcomes (such as rate of development of psychotic illness) than the number of PEs reported (Kline et al., 2014; Loewy et al., 2011).

We also examined whether the benefits of RT were maintained longitudinally. Reductions in both the number of PEs and the degree of associated distress were maintained 12 months after the completion of RT. This could be considered unexpected given the brevity of RT, but it is consistent with other evidence demonstrating potentially long-term benefits of very brief mindfulness-based interventions (Dundas, Thorsheim, Hjeltnes, & Binder, 2016; Mermelstein & Garske, 2015; Walsh, Eisenlohr-Moul, & Baer, 2016). However, future trials with larger samples and additional outcome variables (including those assessing functioning) are needed to provide a better estimate of the longitudinal impact of RT. Also, the fact that the immediate, post-RT improvements in resilience-related skills and reductions in depression and anxiety were not maintained at the 12-month follow-up assessment raises the question of whether increasing the number of sessions or including booster sessions may bolster the long-term effects of the intervention on these symptoms. But this consideration should be balanced with the need to maximize the feasibility of an intervention in a non-treatment seeking population currently enrolled in college (who may be less likely to participate in longer interventions).

In addition, there are several limitations of this study that should be considered when interpreting its results. First, our use of a waitlist control rather than an active control condition does not allow us to draw definitive conclusions regarding the specific efficacy of the RT intervention, but rather represents an intermediate step between our initial one arm study and a full efficacy trial. Second, the limited diversity of this sample with respect to race, gender, and year in college reduces the generalizability of these findings. The program was intentionally enriched for first-year students by design (e.g. by selecting recruitment locations where first-year students spend their time), because we aimed to (1) intervene as early as possible during college, to maximize any reduction of risk for future mental illness associated with RT, and (2) longitudinally follow study participants throughout college. Finally, we chose not to assess hallucinations as a specific, separate outcome, given that delusional thinking is more commonly endorsed by college students than hallucinations (Lincoln & Keller, 2008) and the 21-item PDI (our measure of PEs) includes 7 items assessing common perceptual abnormalities. However, future studies should examine hallucinations in a more comprehensive manner as a possible target of RT.

In summary, these findings show that RT, a brief resilience-boosting, group-based psychological intervention, is associated with a reduction in levels of psychopathology and an increase in resilience-related capacities in the short term, with sustained reductions in subclinical psychotic symptoms and in the distress associated with them 1 year later. This long-term effect of RT on these symptoms may provide some protection against developing a serious mental illness, since the presence of subclinical psychotic symptoms is the most well-established risk factor for developing clinical psychosis over time (Cannon et al., 2008; Dominguez et al., 2011; Kelleher & Cannon, 2011; Loewy et al., 2011; Poulton et al., 2000). Future trials can compare the efficacy of RT to that of other interventions tailored to this age group, in those who have identifiable risk factors. Our results suggest that even at a very early stage, when only mild depressive and PEs are present and individuals are functioning well enough to attend college, these symptoms can be diminished, potentially reducing the overall risk for the development of serious mental illnesses during this peak time of neurodevelopmental vulnerability.

## Supplementary material

For supplementary material accompanying this paper visit https://doi.org/10.1017/S0033291722000046. click here to view supplementary material
