# Supplementary material for: Efficacy of a transdiagnostic, prevention-focused program for at-risk young adults: a waitlist-controlled trial
Source: Psychol Med. 2022 Mar 1;53(8):3490–9. doi: 10.1017/S0033291722000046 (PMC9433469; doi:10.1017/S0033291722000046)
Supplement: Supplementary file 1 [file S0033291722000046sup001.docx]

**Supplementary Materials for DeTore, Luther et al “Efficacy of a transdiagnostic, prevention-focused program**

**for at-risk young adults: A waitlist-controlled trial”**

**Supplemental Methods.**

Descriptions of the Measures.

Depressive symptoms: The Beck Depression Inventory – 1A (BDI – 1A; (Beck, 1961) is a 21-item self-report scale that was used to measure the affective, cognitive, motivational, and somatic symptoms of depression, with higher BDI-1A total scores indicating more depressive symptoms. Internal consistency was good in the randomized sample with α = 0.85.

Psychotic experiences (PE): The self-report Peters Delusions Inventory (PDI; (E. Peters et al., 2004; E. R. Peters et al., 1999)) was used to assess psychotic experiences. The PDI is a 21-item self-report inventory that was designed to measure delusional beliefs and unusual experiences in the general population. For each endorsed PE, participants rate the associated distress, preoccupation, and conviction. Following our prior methods (Burke et al., 2020), we focused on the number of endorsed PE (PDI total score) and the level of associated PE distress reported (PDI-Distress subscale score), with higher scores indicating greater PE and associated distress, respectively. Internal consistency was acceptable to good, with α = .84 for the total PDI score and α = .76 for PDI Distress score in the randomized sample.

Anxiety symptoms: The self-report Spielberger State-Trait Anxiety Inventory (STAI; (Spielberger et al., 1983)) was used to measure how participants currently and generally feel in terms of anxiety-related thoughts and experiences. We focused on the 20-item STAI-state scores, where higher scores indicate more current anxiety. Internal consistency of the STAI-state scores was high (α = 0.93).

Resilience factors

Resilience. The Connor-Davidson Resilience scale (CD-RISC; (Connor & Davidson, 2003)) is a 25-item self-report scale that was used to assess participant’s ability to adapt and cope with significant challenges or stressful events. Higher total scores on the CD-RISC indicate greater resilience. Internal consistency was high (α = .92).

Self-Compassion. The 26-item self-report Self Compassion Scale (SCS; (Neff, 2003)) was used to assess several domains of self-compassion: self-kindness, self-judgement, common humanity, social isolation, mindfulness, and over-identification. The outcome of interest for the current study was the SCS Total score, with higher scores indicating greater levels of self-compassion. For the SCS total score, the internal consistency was high (α = 0.93) in the randomized sample.

Mindfulness. To assess for mindfulness, we used the multidimensional Five Facet Mindfulness Questionnaire (FFMQ; (Baer et al., 2006). The FFMQ assesses the following five mindfulness domains: acting with awareness, describing, nonjudging, nonreactivity, and observing. The total score was used in the current study, with higher scores indicating higher levels of mindfulness. Internal consistency was good (α = 0.82) for the FFMQ total score.

Mentalization. The self-report Interpersonal Reactivity Index (IRI; (Davis, 1983)) was used to assess for a process that is considered to be related to or based on mentalization ability, empathy. Following our prior work (Burke et al., 2020), we focused on the Empathic Concern (EC) and Perspective Taking (PT) IRI subscales. The EC subscale assess affective empathy through measuring feelings of warmth, compassion, and concern for other’s distress, while the PT subscale assess cognitive empathy or the ability to see things from another person’s perspective. Higher subscale scores are indicative of greater affective and cognitive empathy, and internal consistency was acceptable to good in the current sample α = 0.86 for EC and α = 0.76 for PT subscales.

Positive Affect. The self-report Positive and Negative Affect Scale (PANAS; (Watson et al., 1988)) was used to assess positive affect. The positive affect subscale consists of 10 words describing emotions (e.g., interested, inspired, determined), and participants are asked to rate the extent to which they have felt each emotion on a scale from 1 (*very slightly or not at all*) to 5 (*extremely*). In the current study, internal consistency was good (α = .86) for the positive affect subscale.

Supplemental Figure 1. Changes between Baseline and 4-Week Symptom and Resilience Related Outcomes in the Resilience Training (*n* = 43) versus the Waitlist Control (*n* = 45) Conditions

Resilience Training

Waitlist Control


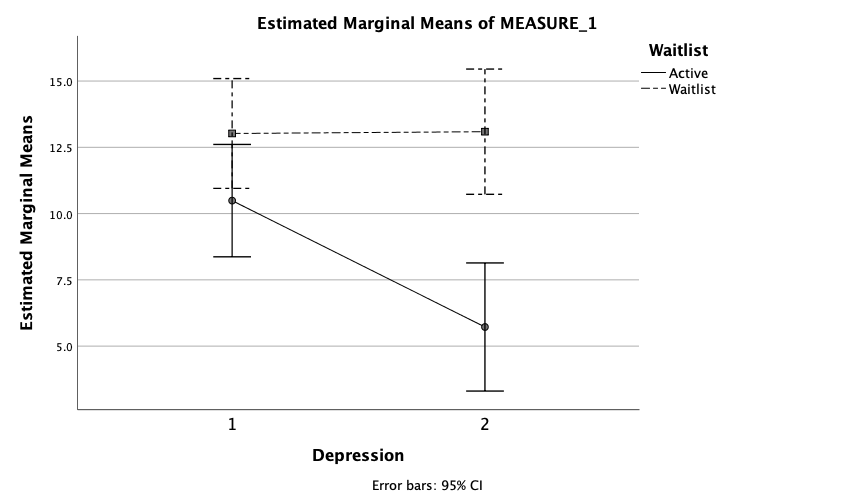


Baseline

Post

Group x time interaction:

*F(1,86) = 13.62, p < .001*

Depressive Symptoms


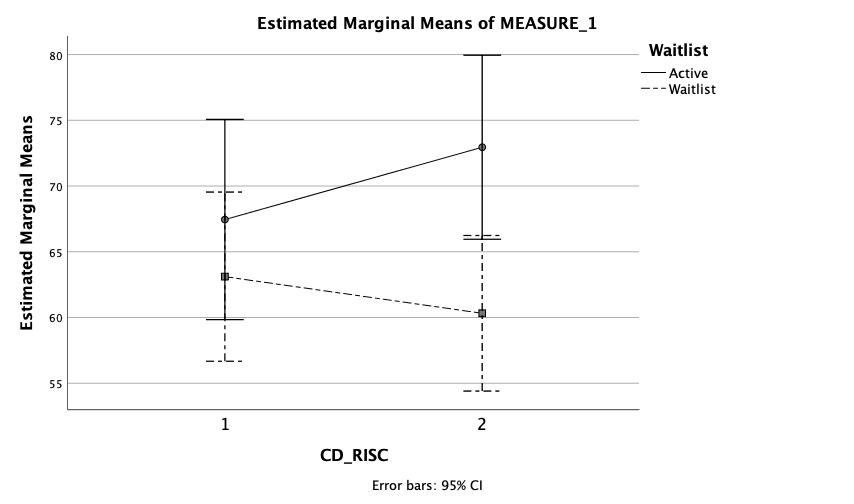


Pre

Post

Group x time interaction:

*F(1,46) = 13.23, p = .001*

Resilience

Pre

Post


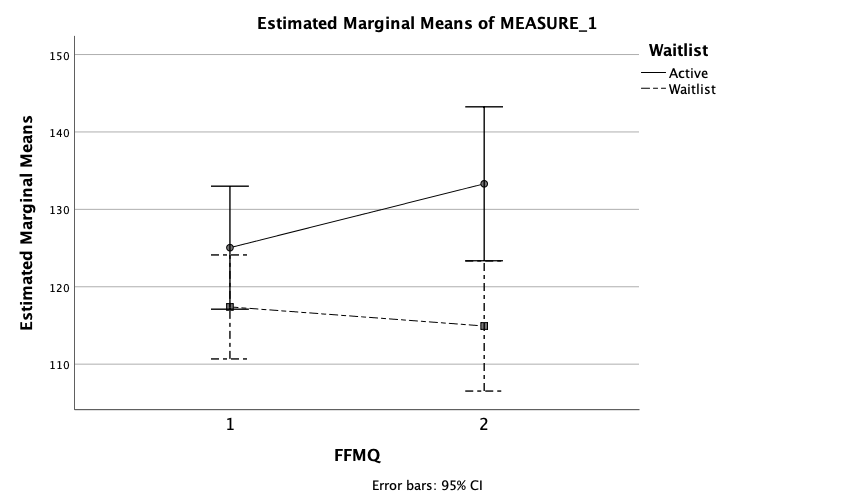


Group x time interaction:

*F(1,46) = 8.32, p = .006*

Mindfulness


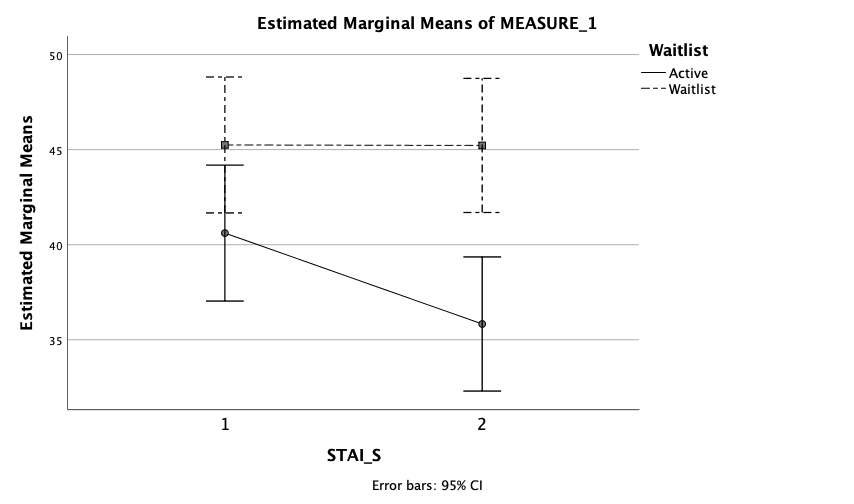


Pre

Post

Group x time interaction:

*F(1,86) = 5.86, p = .02*

State Anxiety


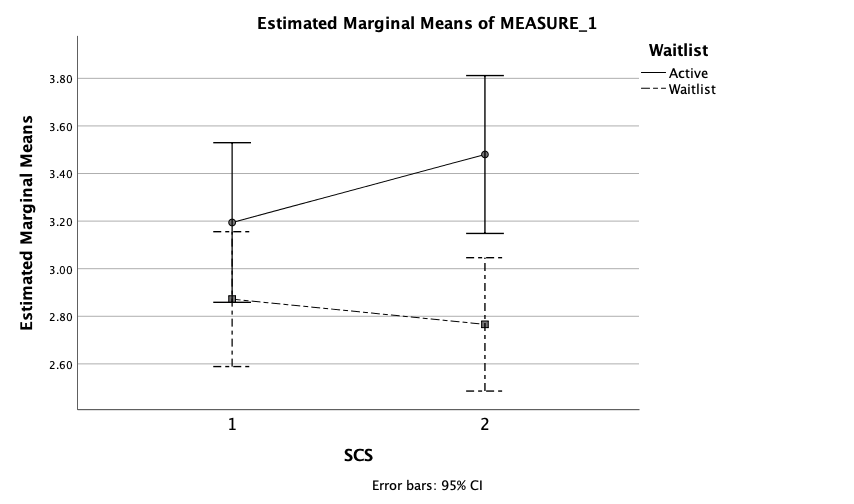


Pre

Post

Group x time interaction:

*F(1,46) = 8.28, p = .006*

Self-Compassion


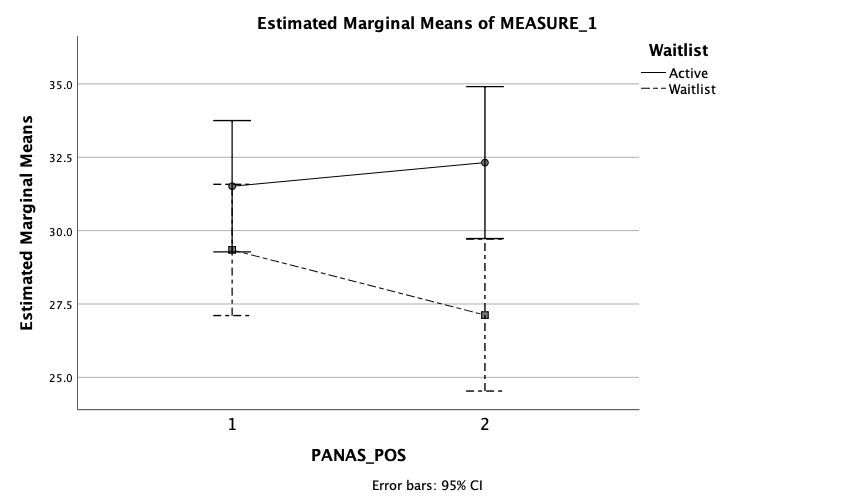


Pre

Post

Group x time interaction:

*F(1,80) = 5.11, p = .03*

Positive Affect

Supplemental Table 1.

Descriptive Statistics and Outcome Changes between Baseline and 4-Weeks for the Resilience Training and Waitlist Groups

|  | Resilience Training | | Waitlist | | Repeated Measures ANOVA Results | | | | | | | | | | | | | |
| --- | --- | --- | --- | --- | --- | --- | --- | --- | --- | --- | --- | --- | --- | --- | --- | --- | --- | --- |
|  | Baseline | 4-weeks | Baseline | 4-weeks | Group Effect | | | | | | Time Effect | | | | Time X Group Effect | | | |
| Measure | M (SD) | M (SD) | M (SD) | M (SD) | *df* | *F* | | *p* | | *η_p_^2^* | *df* | *F* | *p* | *η_p_^2^* | *df* | *F* | *p* | *η_p_^2^* |
| Depression | 10.49 (7.60) | 5.72 (6.69) | 13.02 (6.35) | 13.09 (9.03) | 1,86 | 11.53 | | .001 | | .12 | 1,86 | 12.88 | .001 | .13 | 1,86 | 13.62 | <.001 | .14 |
| PEs | 6.70 (3.45) | 3.40 (2.44) | 7.07 (3.09) | 5.33 (3.30) | 1,86 | 3.73 | | .06 | | .04 | 1,86 | 78.89 | <.001 | .48 | 1,86 | 7.66 | .007 | .08 |
| PE Distress | 17.23 (10.74) | 7.84 (6.84) | 18.78 (10.64) | 14.02 (10.24) | 1,86 | 4.13 | .045 | | .05 | | 1,86 | 69.35 | <.001 | .45 | 1,86 | 7.46 | .008 | .08 |
| Anxiety | 40.61 (11.14) | 35.83 (10.35) | 45.24 (11.85) | 45.22 (12.27) | 1,80 | 9.10 | .003 | | .10 | | 1,80 | 5.99 | .017 | .07 | 1,80 | 5.86 | .018 | .07 |
| Resilience | 67.45 (16.72) | 72.95 (14.01) | 63.11 (17.05) | 60.32 (16.56) | 1,46 | 3.38 | .07 | | .07 | | 1,46 | 1.42 | .24 | .03 | 1,46 | 13.23 | .001 | .22 |
| Mindfulness | 125.05 (19.52) | 133.30 (27.18) | 117.39 (16.21) | 114.93 (17.65) | 1,46 | 5.50 | .02 | | .11 | | 1,46 | 2.43 | .13 | .05 | 1,46 | 8.32 | .006 | .15 |
| Self-  Compassion | 3.19 (.81) | 3.48 (.74) | 2.87 (.70) | 2.77 (.73) | 1,46 | 6.33 | .015 | | .12 | | 1,46 | 1.75 | .19 | .04 | 1,46 | 8.28 | .006 | .15 |
| Empathetic  Concern | 20.00 (4.75) | 19.65 (4.76) | 19.79 (5.50) | 18.59 (5.82) | 1,47 | .20 | .66 | | .004 | | 1,47 | 1.87 | .18 | .04 | 1,47 | .57 | .46 | .01 |
| Perspective  Taking | 19.50 (4.72) | 18.55 (6.35) | 17.79 (4.20) | 16.76 (5.47) | 1,47 | 1.69 | .20 | | .04 | | 1,47 | 2.19 | .15 | .04 | 1,47 | .004 | .95 | .00 |
| Positive  Affect | 31.51 (7.70) | 32.32 (8.38) | 29.34 (6.66) | 27.12 (8.28) | 1,80 | 5.41 | .02 | | .06 | | 1,80 | 1.12 | .29 | .01 | 1,80 | 5.11 | .03 | .06 |

| Supplemental Table 2.  Descriptive Statistics and Pre-Post Resilience Training Outcomes for the Waitlist Group | | | | | | | |
| --- | --- | --- | --- | --- | --- | --- | --- |
|  | Pre-RT | Post-RT | Paired *t*-test Results | | | | |
| Measure | M (SD) | M (SD) | *Df* | *t* | *p* | *d* | |
| Symptoms | | | | | | | |
| Depression Symptoms | 12.74 (8.59) | 8.83 (8.96) | 34 | 5.60 | <.001 | | -.95 |
| Psychotic Experiences | 5.62 (3.46) | 3.82 (2.80) | 33 | 4.64 | < .001 | | -.83 |
| Psychotic  Experiences— Distress | 14.82 (10.66) | 9.44 (9.08) | 33 | 4.17 | < .001 | | -.73 |
| Anxiety Symptoms | 44.65 (11.99) | 39.91 (12.38) | 33 | 3.57 | .001 | | -.61 |
| Resilience Factors | | | | | | | |
| Resilience | 62.21 (10.43) | 65.96 (13.72) | 23 | -2.24 | .04 | .49 | |
| Mindfulness | 113.83 (12.07) | 120.17 (17.98) | 23 | -2.16 | .04 | .47 | |
| Self-Compassion | 2.73 (0.50) | 3.06 (0.61) | 23 | -2.51 | .02 | .52 | |
| Empathetic Concern | 18.75 (5.67) | 17.96 (5.55) | 23 | 1.32 | .20 | -.27 | |
| Perspective Taking | 16.71 (5.86) | 17.33 (5.42) | 23 | -.65 | .52 | .13 | |
| Positive Affect | 27.32 (6.82) | 30.74 (6.79) | 33 | -3.98 | <.001 | .68 | |
| *Note.* RT = Resilience Training; WL = Waitlist. | | | | | | | |

| Supplemental Table 3.  Descriptive Statistics and 12-Month Outcomes for Resilience Training across both the RT and WL Groups | | | | | | | |
| --- | --- | --- | --- | --- | --- | --- | --- |
|  | Pre-RT | 12-months | Paired *t*-test Results | | | | |
| Measure | M (SD) | M (SD) | *df* | *t* | *p* | *d* |  |
| Symptoms | | | | | | |  |
| Depression Symptoms | 12.81 (7.69) | 13.53 (10.98) | 41 | -.45 | .66 | .07 |  |
| Psychotic Experiences | 6.90 (3.01) | 4.10 (3.29) | 41 | 5.93 | <.001 | -.92 |  |
| Psychotic Experiences—  Distress | 18.67 (10.66) | 11.62 (12.98) | 41 | 4.53 | <.001 | -.71 |  |
| Anxiety Symptoms | 44.97 (10.56) | 42.39 (11.46) | 37 | 1.44 | .16 | -.23 |  |
| Resilience Factors | | | | | | |  |
| Resilience | 64.24 (15.60) | 64.24 (18.00) | 33 | .000 | 1.00 | .00 |  |
| Mindfulness | 118.38 (16.90) | 122.00 (21.00) | 33 | -1.50 | .14 | .27 |  |
| Self-Compassion | 2.95 (0.61) | 2.98 (0.64) | 35 | -.28 | .78 | .05 |  |
| Empathetic Concern | 19.24 (6.11) | 17.65 (5.66) | 36 | 1.95 | .06 | -.32 |  |
| Perspective Taking | 17.62 (3.95) | 17.05 (4.20) | 36 | .81 | .42 | -.13 |  |
| Positive Affect | 28.79 (6.88) | 27.76 (7.79) | 37 | .89 | .38 | -.15 |  |

*Note.* RT = Resilience Training; WL = Waitlist.
